# Reframing PTSD for computational psychiatry with the active inference framework

**DOI:** 10.1080/13546805.2019.1665994

**Published:** 2019-09-30

**Authors:** Adam Linson, Karl Friston

**Affiliations:** a Faculty of Natural Sciences & Faculty of Arts and Humanities, University of Stirling, Stirling, UK; b Wellcome Centre for Human Neuroimaging, UCL, London, UK

**Keywords:** Post-traumatic stress disorder (PTSD), psychopathology, audition, embodiment, evolution

## Abstract

**Introduction:** Recent advances in research on stress and, respectively, on disorders of perception, learning, and behaviour speak to a promising synthesis of current insights from (i) neurobiology, cognitive neuroscience and psychology of stress and post-traumatic stress disorder (PTSD), and (ii) computational psychiatry approaches to pathophysiology (e.g. of schizophrenia and autism).

**Methods:** Specifically, we apply this synthesis to PTSD. The framework of active inference offers an embodied and embedded lens through which to understand neuronal mechanisms, structures, and processes of cognitive function and dysfunction. In turn, this offers an explanatory model of how healthy mental functioning can go awry due to psychopathological conditions that impair inference about our environment and our bodies. In this context, auditory phenomena—known to be especially relevant to studies of PTSD and schizophrenia—and traditional models of auditory function can be viewed from an evolutionary perspective based on active inference.

**Results:** We assess and contextualise a range of evidence on audition, stress, psychosis, and PTSD, and bring some existing partial models of PTSD into multilevel alignment.

**Conclusions:** The novel perspective on PTSD we present aims to serve as a basis for new experimental designs and therapeutic interventions that integrate fundamentally biological, cognitive, behavioural, and environmental factors.

## Introduction

Post-traumatic stress disorder (PTSD) affects a significant segment of society, including combat veterans, first responders, sufferers of childhood and domestic abuse, and many others who have endured traumatic experiences ranging from terror attacks to severe transportation and industrial accidents. PTSD lowers the quality of life of those with the condition and those around them, and can even precipitate perilous situations during acute episodes. While PTSD has gradually come to be regarded as a collection of heterogeneous conditions, with a variety of disparate causes and effects, there are also many promising syntheses of previous studies and models. Yet, such syntheses tend to be relatively monolithic in their disciplinary scope, focusing exclusively on (e.g.) neurobiology or behavioural psychology.

Here, we present a novel view of PTSD that unifies reviewed work in neuroscience and psychology using the active inference framework. The (meta) theoretical contribution of this paper is just to provide a first principle account of extant formulations—so that they can be seen in light of each other. We look at neuropsychological dysfunction, both generally and in relation to PTSD. We argue that understanding the aetiology of PTSD requires the recasting of some traditional “classical paradigm” notions of healthy cognitive function—executive control, attention, contextual processing—in active inference terms (for an introduction, see e.g., Friston, 2009; for relevant details, see especially Brown, Friston, & Bestmann, 2011; Feldman & Friston, 2010; for background on the present context, see also Linson, Clark, Ramamoorthy, & Friston, 2018).

Broadly, our approach builds on the success of current research in computational psychiatry. Our emphasis here is on “reframing” PTSD, to contextualise extant formulations in relation to our subsequent computational work. This foundational theoretical treatment is motivated by the difference our approach entails from the classical information processing scheme (in which environmental “input” is neurally “computed”). In particular, we specify the abstractions required for modelling body/environment equilibrium-maintaining behaviour via a sensorimotor nervous system.^1^

PTSD symptoms related to memory, arousal, and mood (DSM-5) have been the subject of earlier computational models (for a review, see Radell, Myers, Sheynin, & Moustafa, 2017). In contrast, our emphasis lies in showing how adaptive/healthy behaviour can become impaired, leading to maladaptive/pathological behaviour (see e.g., Sherin & Nemeroff, 2011). This approach seeks to construe PTSD as resulting from a quasi-lesion to a well-functioning system, and hence, an emergent phenotype, as opposed to a phenotype posited post-hoc on the basis of symptoms.

The conceptual treatment of PTSD in this paper represents a variant of computational psychiatry that is not rooted in the analysis of quantitative cohort data. Rather, our aim is to develop a generative model based on active inference (Friston, FitzGerald, Rigoli, Schwartenbeck, & Pezzulo, 2016) that can be realised in terms of message passing and belief updating—and accompanying electrophysiological and behavioural responses (Parr & Friston, 2018). In brief, we try to show how phenomena that emerge from first (Bayesian) principles relate to clinical constructs (and the opportunity for *in silico* experiments). In this paper, we appeal to empirical studies to demonstrate the construct validity of an active inference account of PTSD. In subsequent work, we hope to use this account to explain electrophysiological and psychophysical responses that characterise PTSD (Linson, Parr, and Friston, in review).

## Explanatory stack

Our reframing of PTSD is built on an explanatory “stack” of four *interacting* levels that comprise the explanation^2^: (i) an embodied-embedded level, regarding evolutionarily, developmentally, and situationally constrained body/environment behavioural dynamics; (ii) a neurobiological substrate, ranging from low-level sub-cellular mechanisms to high-level functional neural regions; (iii) a hierarchical Bayesian model of body/environment interactions, in terms of priors, hypotheses, and evidence; and, (iv) a psychophysical / phenomenological level, in terms of experimental observation and self-report related to perceptual sampling and associative machinery.

Here, we give a brief rundown of how our explanatory stack is applied to PTSD, beginning with the active inference formulation of reward. Under active inference, reward is recast as minimising the expected free energy following an action (Friston, 2010; Friston et al., 2016), while embodied action is recast as proprioceptive inference.^3^ An agent that actively brings about its own future implicitly takes into account both metabolic expenditure (expected caloric investment and burn rate) and the lag-time in disambiguating sensory evidence as well as in realising a possible action. Thus, plausibly selected-for evolutionary hyperpriors and hyperparameters (i.e., the embodied-embedded architecture) pertaining to organism and species survival would contribute to the governing of action selection in light of counterfactual trade-offs in metabolic and temporal costs (Linson et al., 2018).

More concretely, as an example of a merited high, rapid caloric expenditure, we consider a situationally apt “survival” prior that underwrites a “fight or flight” policy. This prior is based on two simplifying assumptions. The first is that survival and comfort are circumstantially malleable, biologically inherited (i.e., selected for) “default” preferences over death and anxiety (facilitating sufficient reproduction). The second is that the designation “fight or flight” suffices to pick out the conditions that require a high metabolic rate in the motor system, a focal point of the present analysis, along with a “freeze” state, with a similar neuronal footprint, but combined with motor inhibition. Generally, we acknowledge that this formulation does not capture the full range of responses to perceived threat (Bracha, Ralston, Matsukawa, Williams, & Bracha, 2004; LeDoux, 2012; Roelofs, 2017). Complexities omitted from this abstraction include indications that the serial choice order of behavioural alternatives appears to be flight, when possible, and fight when flight is not possible. In addition, beyond the “freeze” state, which we consider later in relation to hypervigilance and avoiding detection, there is also thought to be a “fright” state (alternatively known as panic, tonic immobility, and “playing dead”), regarded as a survival mechanism during a commenced threat engagement (beyond the present scope).

A survival prior that selects a “fight or flight” policy would mandate a high metabolic expenditure (expected caloric investment and burn rate) in the motor system, following active inference characterisations of attention and motor preparation (Brown et al., 2011; Feldman & Friston, 2010). Under this policy, given fundamental energetic constraints, animals including humans would plausibly deploy metabolic expenditure to (near-field) threat-engaging “fight” behaviour or (near- or wide-field) threat-aversive “flight” behaviour, such that energy is diverted from exploratory sensing and awareness of a global threat scene (see Mirza, Adams, Mathys, & Friston, 2016). In this case, to ensure its survival, the organism must resist returning to a “resting” policy until the perceived threat is neutralised or has subsided—to a sufficient degree of certainty. And yet, if all the organism’s energy is expended, it will forcibly return to a resting state irrespective of the threat (i.e., in safe or unsafe conditions). To strike a balance in favour of survival, the “fight or flight” policy should include an occasional minimal scene resampling, to survey the possibility that it may be safe to return to a resting state. Throughout the paper, we continue to flesh out this key example with finer-grained details and supporting evidence.

In what follows, the account of PTSD we develop focuses on a maladaptive response to stress from an evolutionary perspective, while otherwise sharing much in common with the comprehensive general treatment of stress by Peters, McEwen, and Friston (2017). Similarly, our view of the relationship between evolution and neural circuits relevant to PTSD is closely aligned with a broader account of emotion by LeDoux (2012). More generally still, our notions of embodied and embedded can be understood in relation to the idea that self-preservation amounts to the maintenance of bodily and mental well-being in the face of threats external and internal to the body/environment boundary (Peters et al., 2017).

In short, for any creature, self-preservation amounts to bringing about a future in which its continued self is underwritten by the avoidance of existential danger, which can be understood as a process of active inference. This points to a difficult (implicit) problem in the face of an approaching or acute threat: how should my metabolic resources be allocated to ensure my own protection? We consider how animals such as humans embody a solution (via natural selection) that is generally effective in health and that goes awry in PTSD, with consequences that can be linked to its known physical and psychological manifestations.

## Evolution and active inference: ecologically situated perception

Animal studies on PTSD have considered the evolutionary continuity from invertebrates to vertebrates, in relation to their adaptive responses to life-threatening psychological stressors and associated stimuli (Clinchy et al., 2011). These responses become maladaptive when they induce sustained physiological effects beyond the threat condition. This evolutionary continuity can be considered more generally in terms of organismic biophysical architectures (Niven, 2016; Niven & Laughlin, 2008). Consider, for example, that for food-deprived blowflies, metabolic resources dedicated to motor control remain relatively constant, while the expenditures needed for exteroceptive sensing are significantly throttled (Longden, Muzzu, Cook, Schultz, & Krapp, 2014). More generally, across species, greater energy expenditure is required for higher throughput from the sensory system to the nervous system (Niven, Anderson, & Laughlin, 2007). On the other hand, a brain leveraging dynamical instability (i.e., self-organised criticality) can propagate stimulus-evoked transients more efficiently, a state associated with gamma frequency rhythms (Robinson, 2006). The significance of this for PTSD will become apparent below.

Notably, for organisms with spatially extensive niches, due to how sound travels through space, auditory stimuli can be more relevant than other sensory modalities, and acoustic cues can be more frequently alarming (Clinchy, Sheriff, & Zanette, 2013). It is also notable that, in the evolutionary biodiversity record, an inverse correlation has been identified between the narrowness of foveal focal range (the width in degrees of sharpness in the visual field) and auditory localisation precision (Heffner, 2004). The record shows that, across a wide range of mammals, humans rank among the narrowest field of sharp vision, near 1°, and also the greatest precision in locating (the azimuth of) an auditory stimulus in a 360° egocentric field. In terms of biophysics, it can also be noted that, in humans, the auditory pathway from the sensory surface to the top of the cortical hierarchy responds more rapidly and with more reliably low-latency stimulus-locked neuronal correspondences than the visual system (Kopp-Scheinpflug & Tempel, 2015). In addition, psychophysical experiments have shown that, in the face of complex auditory interference (known as informational and energetic masking, revisited in the next section), auditory discrimination of a target is enhanced by visual cueing of the target location (Best, Ozmeral, & Shinn-Cunningham, 2007).

Given these premises, we can focus in on the critical significance of multisensory integration in reducing uncertainty about potentially self-endangering distal stimuli (e.g., predators). Consider a scenario in which a human subject hears an unidentified sound from behind. In the first instance, the sound is a surprisal (in the information-theoretic sense) that indicates physical energy in the environment, beyond visual range. Since discovering the source of this energy could be vital (in case it is an existential threat), the surprisal must be reduced.

We can describe two plausible surprisal-reducing responses: the first is through the process of active inference, that would direct biomechanical movement (e.g., of head, neck, and torso) in such a way that brings the stimulus source into the narrow foveal field. At this point, visually foraged sensory information may reduce the surprisal by “filling in the blanks” (Friston et al., 2017). Namely, saccades lead to resolving uncertainty about the “hidden” cause of the stimulus. This informational or epistemic foraging (i.e., active sensing) will be selected if it reduces expected surprisal (i.e., uncertainty) above and beyond the surprisal associated with a costly re-orienting response. This form of active inference samples salient information that propagates upwards through the cortical hierarchy, to update a generative model of the scene at the level of familiar spatiotemporal entities such as objects and animals (Mirza et al., 2016).

A second and equally valid response would be to treat the sound as indicative of an existential threat—in other words, to treat the sound as evidence for a “threat” hypothesis—that would entail a reactive, motor-intensive “fight or flight” policy. The latter would not require immediate sensory confirmation from the environmental scene. Instead, metabolic resources would be diverted from cortical propagation to the limbic system, especially to the locomotor and sympathetic nervous system (see Longden et al., 2014; see also Laughlin, de Ruyter van Steveninck, & Anderson, 1998). This adaptive threat response would be naturally selected for, as nascent species that reduced stimulus uncertainty at the expense of reducing uncertainty about their own safety would fail to ensure self-preservation in the face of actual existential endangerment.

## Audition

In addition to the above evolutionary considerations, we focus on audition for two related reasons. The first regards the well-studied role of auditory phenomena in PTSD (e.g., the acoustic startle response), and the second regards the application of the explanatory stack to auditory functioning in well-known paradigms (the “White Christmas test” and “cocktail party effect”, considered below). The illustration of what can go wrong in healthy auditory function in turn supports the argument we develop with respect to PTSD. This does not imply that the auditory system is inherently tied to PTSD; rather, that—in ecologically valid contexts—auditory sensation tends to have greater ambiguity (i.e., higher entropy) than visual sensation, which provides an entry point into our model.

In an experimental study by Shalev et al. (2000), PTSD sufferers responded adversely to an auditory trigger; the study’s authors note that “it seems implausible that the simple tone stimulus employed [in the study] could have been associated with the various traumatic events experienced by the subjects because of its generic nature”, consistent with the present proposal for a strong “threat” prior (in connection with what we describe below as an underfitting scenario). Shalev and colleagues suggest that the adverse response might reflect “an impaired capacity to correctly classify intense, yet redundant, auditory stimuli as harmless”. On our corresponding picture with a strong threat prior, the impairment would relate to a diminished capacity to resample the scene and update beliefs about the actual state of affairs on the basis of new information (cf. Chalk, Seitz, & Seriès, 2010; Sotiropoulos, Seitz, & Seriès, 2011), which is metabolically expensive.

**Figure 1.**
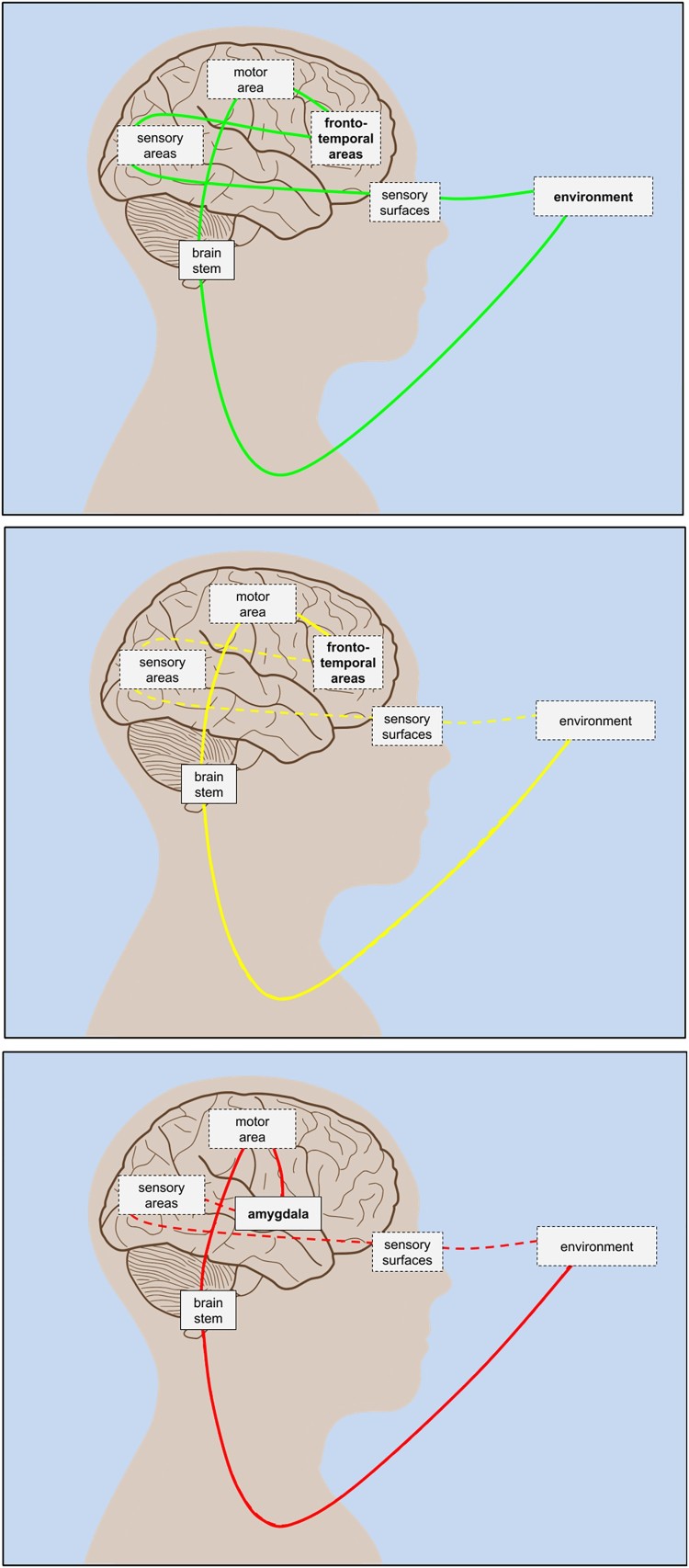
Simplified schematic model of active inference sensorimotor loop. Solid lines indicate strong / dense correspondence (high mutual information), dashed lines indicate weak / sparse correspondence (low mutual information). Bold typeface indicates modulatory influence driving propagation. Lines passing through depicted brain regions indicate primary relevant (bidirectional) pathways. *Top panel:* Healthy function. Full loop through environment-body-brain system joins traditional top-down and bottom-up directions with balanced agent (frontotemporal) / environment modulatory coupling. *Middle panel:* Psychosis-related dysfunction. Functional decoupling between traditional top-down and bottom-up pathways; impairment in traditional “reality testing”. *Bottom panel:* Stress-related dysfunction (e.g., PTSD). Hypofrontal amygdala activation in “fight or flight”.

Our account underscores a continuous loop through the brain that stabilises the body in its environment (Figure 1a), which amounts to having a “grip” on situational coordination. This reframes the traditional notion of impaired “reality testing” as a strong top-down bias that is (atypically) decoupled from bottom-up confirmation (Figure 1b). Crucially, on this view, top-down biases have a profound effect on the way sensory evidence is garnered to confirm or disconfirm these biases. In other words, the deficit we explore in this treatment rests upon inferential biases in the brain that preclude the gathering of the sensory evidence which would challenge them, i.e., a failure of “reality testing” due to “biased sampling”. From this perspective, since the environment continues to be sampled, external reality does not remain untested; and, while bias is often regarded as problematic, it can be adaptive or maladaptive, depending on the circumstances.

This overall picture is consistent with evidence showing that, for those with a tendency toward psychosis, simple ambiguous stimuli (that presumably require less high-order cortical predictive dynamics) are less likely to evoke an attribution of an “unpresented” signal in noise; in contrast, complex ambiguous stimuli elicit more false positives (Grant, Balser, Munk, Linder, & Hennig, 2014), suggesting that high-order cortical activation can become a “runaway” top-down process, decoupled from further bottom-up confirmation (Figure 1b). In an auditory context, those highly disposed toward hallucination are more likely to believe they heard a sound that was not presented (Bentall & Slade, 1985). Conversely, a well-functioning continuous action-perception loop—equivalent to unimpaired reality testing—corresponds to maintaining an appropriate sensitivity to sensory evidence, via stimulus-locked neuronal activity, such as real-time high-order comprehension of a complex auditory scene (Plakke & Romanski, 2014).

While this picture of healthy function and dysfunction largely corresponds to healthy and impaired classical top-down executive control, the classical description risks underestimating the role of environmentally embedded, embodied sensorimotor loops. For instance, Arnsten (2009) presents compelling evidence on low-level mechanisms and pathways concerning stress-related biological impairment, described in terms of a mutually inhibitory trade-off between “thoughtful ‘top-down’ control by the PFC” and “‘bottom-up’ control by the sensory cortices”. Remaining consistent with the underlying evidence, the picture can be repainted in such a way that explains PFC-engagement not as top-down executive control, but rather in terms of a how the brain samples the environment, specifically, how it calibrates the sampling of—and sensitivity to—sensory feedback. This sampling can be overt, as in the saccadic sampling of visual information, or covert, as in setting the precision of ascending sensory signals via attentional selection (Feldman & Friston, 2010). In either case, a proper closure of the action-perception loop rests upon a delicate and carefully orchestrated exchange between the sensory system and the PFC (Figure 1a).^4^

When this coordination is inhibited, we can identify a correspondence to the notion of “underfitting” in machine learning that relates to low variance in sensory feedback. This is again consistent with the low-level mechanisms and pathways described in Arnsten (2009), while not well-captured by the classical description of bottom-up sensory control. Instead, we view the stress-induced impairment as one in which epistemic foraging behaviour is subverted by prior beliefs, which amounts to a functional decoupling between the systems responsible for exteroceptive inference (e.g., in the hippocampal-prefrontal pathway; Godsil, Kiss, Spedding, & Jay, 2013), and those responsible for motor (proprioceptive) and autonomic (interoceptive) functioning. Hence, in the stress-impaired condition, exteroceptively informed scene recognition becomes functionally decoupled from the higher-level cortical dynamics that would normally deploy auditory or visual information gathering (Figure 1b). Phenomenologically, this manifests as misrecognition, delusion, or hallucination (i.e., false inference), driven by associations that may nevertheless continue to correspond to sparse sensory samples (Silbersweig et al., 1995).

### Auditory phenomena

A sort of auditory hallucination—that is not necessarily unhealthy—can be found in the “White Christmas test”, in which subjects that have been verbally primed with the association of a familiar song hear the (unpresented) song during white noise stimulus presentation (Barber & Calverley, 1964). This is akin to the underfitting scenario described above, in which the strong prior is ultimately harmless. Notably, however, fantasy-prone subjects exhibit this misattribution effect in greater proportion than the general population (Merckelbach & van de Ven, 2001; van de Ven & Merckelbach, 2003). There is a significant overlap between individuals with fantasy proneness and psychosis proneness—especially when fantasy arises as a mechanism for mental escape from traumatic circumstances (Rhue & Lynn, 1987). It is therefore not surprising that PTSD and psychosis have a high rate of comorbidity (Hamner, Frueh, Ulmer, & Arana, 1999; see also Braakman, Kortmann, & Van Den Brink, 2008).

Another widely studied auditory phenomenon, the “cocktail party effect” (Cherry, 1953), can be cast in terms of model competition under strong priors (Feldman & Friston, 2010). Depending on the priors and the corresponding model selection, this can lead to both healthy and unhealthy behavioural responses. For example, if you mistakenly believe you heard your name called, and wish to interrupt your present conversation to focus on the hypothesised source, this may not rise beyond the level of mild impoliteness. However, if you mistakenly believe that you overheard a threatening comment directed at you, and you elect to respond with physical violence, it would be reasonable to classify this as a dysfunctional behaviour. Formally, these scenarios would be identical, and yet, a meaningful difference emerges when linking them to socially embedded behaviour. Furthermore, if an ambiguous stimulus easily evokes a strong threat prior—i.e., easily recruits an internal hypothesis associated with a past threat—this would produce the psychophysical and behavioural hallmarks of PTSD.

A further connection to auditory research can be drawn from the distinction between informational and energetic masking. Here, we connect these masking phenomena to healthy function, and later, we tie them to PTSD. In auditory cognition studies, informational masking refers to a scenario in which two simultaneous acoustic signals reach the sensory system, but only one reaches the threshold of perceptual awareness—thought to be a semantic interference effect (i.e., within the auditory hierarchy). This is understood as distinct from energetic masking, in which one acoustic signal prevents a competing signal from discernible sensory surface perturbation. Informational masking amounts to what could be considered involuntary selective attention to a non-target stimulus, whereas energetic masking amounts to what could be considered the “overwriting” of a target stimulus signal. While both phenomena are typically studied in austere controlled experiments, an ecologically valid example might be failing to comprehend a conversation partner due to either (a) a quieter nearby conversation that distracts and inhibits comprehension (informational masking) or (b) loud music playing, that precludes hearing speech in the first place (energetic masking).

Given the above characterisation, the Feldman and Friston (2010) approach to selective attention would explain informational masking as a case of treating an alternative stimulus (even a quieter one) as more salient (i.e., uncertainty resolving) than a current (i.e., predictable) target stimulus, thereby requiring a higher-level hypothesis about “what I am listening to” to be selected, in order to attend to the more salient source. This higher-level policy would be in competition with the policy that maximises evidence for the (predictable) target, and this competition is subsumed within a hierarchy that, at a higher level still, may include an awareness threshold for subjective perception (Friston, 2008; Friston, FitzGerald, Rigoli, Schwartenbeck, & Pezzulo, 2016). In predictive coding terms, this form of attentional selection is thought to be mediated by top-down control of neuromodulatory mechanisms that gate ascending prediction errors (i.e., sensory information).

Thus, in the healthy, full loop through the environment (Figure 1a), the attended to sensory information propagates to higher levels of the cortical hierarchy to revise prior beliefs. Here, beliefs are understood as implicit and, in computational terms, subsymbolic, which underpin explicit beliefs (that can be expressed symbolically), as in self-report during psychophysical experiments. This hierarchical model of selective attention is consistent with evidence from direct cortical recordings showing that, along with attended to speech, ignored speech is tracked in low-level auditory cortices, while in higher-order regions, only the attended to speech is tracked (Zion Golumbic et al., 2013). It is also consistent with evidence from psychological experiments showing that people can more effectively track (or ignore) a familiar voice than an unfamiliar one in the presence of an interfering voice (Johnsrude et al., 2013).

While energetic masking is regarded as a separate phenomenon, we can collapse the energetic-informational dichotomy on the basis of evolutionary criteria, using the active inference framework. Namely, it is plausible to infer that an unexpected high-energy stimulus is a salient indication of something worth tracking in the environment, such as a possible existential threat; support for this is also suggested by empirical studies of auditory response in the amygdala (Bordi & LeDoux, 1992). Unexpected threat signals (rather than expected ones) have been found to evoke startle responses in a manner specific to PTSD sufferers (Grillon et al., 2009), although this alone is insufficiently diagnostic (as it occurs in other psychopathological conditions as well). This reaction may be related to imbalanced neurochemical modulation of the arousal system by norepinephrine signalling, invoked by unexpected uncertainty, which can be distinguished from cholinergic modulation by expected uncertainty or precision (Feldman & Friston, 2010; Yu & Dayan, 2005).

**Figure 2.**
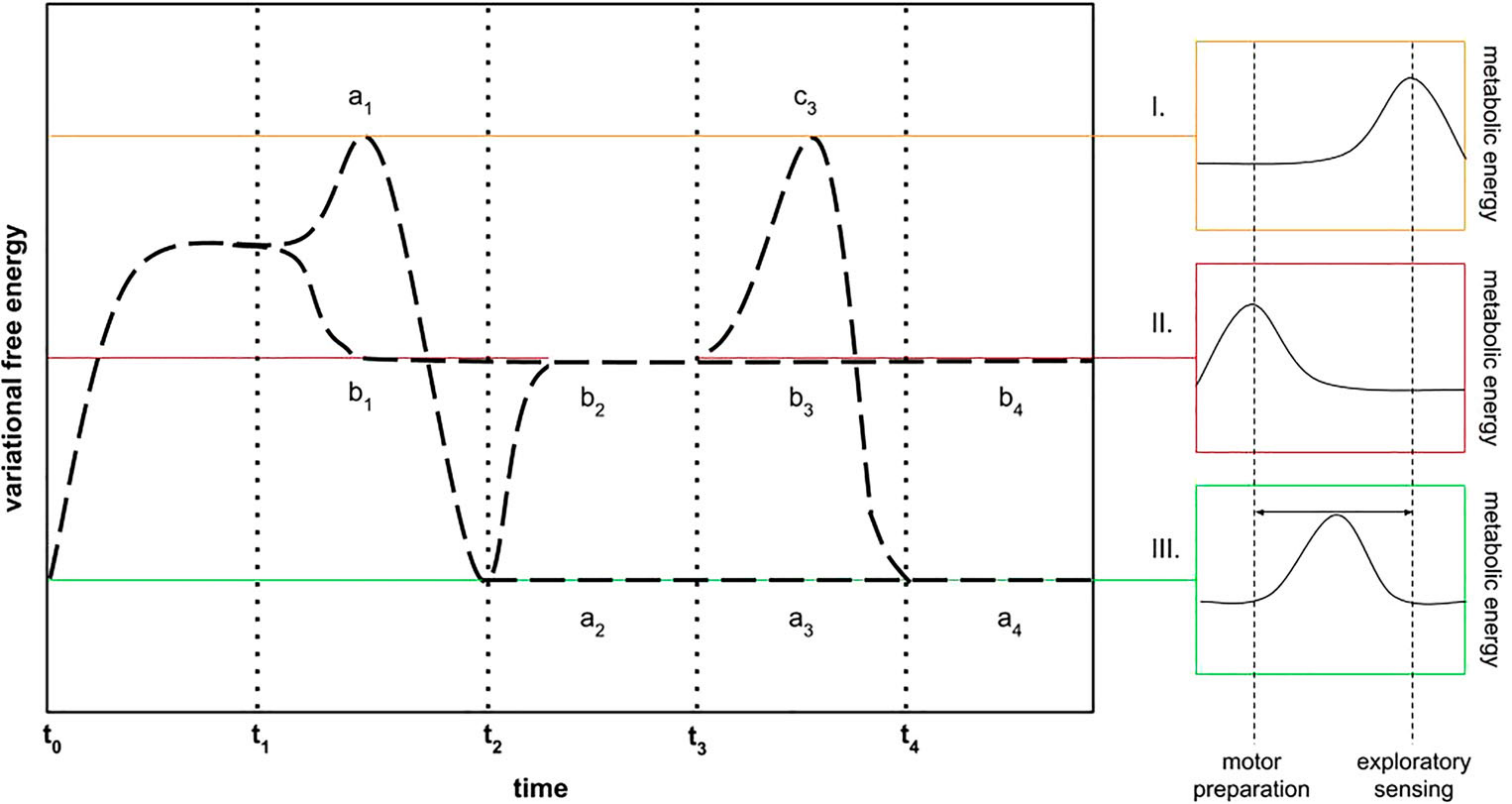
Variational free energy and metabolism in health and with PTSD impairment. (I) Threat or safety undetermined (high uncertainty average, high variance / exploratory sensing, i.e., environment-centric attention). (II) Threat determined (low uncertainty average, low variance / motor preparation, i.e., ego-centric attention). (III) Safety determined (low uncertainty average, high variance / dynamic attentional allocation). The dotted line at *t*_0_–*t*_1_ depicts initial surprisal by a high-energy auditory stimulus outside of foveal range. A healthy individual may engage in exploratory sensing (a_1_) with momentary high uncertainty, and obtain fine-grained stimulus information at t_2_. If no threat is detected, a safety condition with low uncertainty is designated, with fluid, flexible, reality testing, as self-evidencing environment-body-brain dynamics (see Figure 1a), continuing in a_2-4_. If a threat is detected, metabolic resources are directed away from exploratory sensing toward motor preparation (b_2_), with intermittent exploratory sensing to ascertain if the threat is no longer present (c_3_), in which case, a_4_ is reached. Most of the same line may be followed if the healthy person immediately classes the ambiguous stimulus as a threat (b_1_), continuing as with the previous case to b_2_, c_3_, and a_4_. In contrast, PTSD impairs both the a_1_ and c_3_ trajectories, such that the surprising stimulus is disproportionately classed as a threat (b_1_), and further exploratory sensing is not permitted to escape the threat condition in b_2_, continuing instead to b_3–4_ (see Figure 1c).

Using the example of an unexpected high-energy stimulus, and following our characterisation of a “threat” prior, such a stimulus would be informationally (i.e., semantically) relevant, thereby collapsing energetic and informational masking into *hypothesised situational salience*. Under healthy conditions, a possible threat may merit further scene exploration, such as turning toward the high-energy sound source, using active inference to reduce surprisal, as depicted in Figure 2, a_1_. Under impaired conditions, model competition for maximising threat evidence would plausibly result in a “fight or flight” policy selection that would amount to diverting attention to the motor system (Brown et al., 2011), as depicted in Figure 2, b_1_.

For an alternative perspective on what has traditionally been regarded as “generalisation of conditioned fear” (Lissek et al., 2008), we can instead regard the same phenomenon as a forcibly limited disambiguation of a threat-associated stimulus, which is rapidly classed as a threat, precluding any “reality testing”. This amounts to a lowering of the threshold of evidence accumulation for a threat. The limitation is underwritten by a failure to engage in further epistemic foraging, i.e., exploratory sensing and/or entertaining competing (implicit) hypotheses. In other words, with impairments such as PTSD, competing policies for resolving uncertainty—by minimising expected surprisal—are rejected in favour of pragmatic “fight or flight” policies (Figure 2, b_1_), or a “freeze” policy, which we consider below.

This is consistent with findings suggesting that patients with panic disorder designate a stimulus as a threat (i.e., become fearful) for stimuli that differ to a greater degree from a conditioned fear stimulus than such designations by healthy subjects. In other words, healthy subjects typically require a stimulus that is sufficiently similar to a conditioned fear stimulus to become fearful, suggesting that dysfunction reduces disambiguation (discrimination) capacity (Lissek et al., 2010), which is another way of understanding a stimulus gradient. Further support is found in a study showing that training to improve the perceptual discrimination of danger- and safety-associated ambiguous stimuli (i.e., “safety learning”) corresponds in a reduction of the fear generalisation phenomenon (Lommen et al., 2017). Similarly, our perspective supports the recasting of “fear extinction”, often studied in relation to PTSD, as a reduction in the biased disambiguation of a stimulus as a threat, rather than a non-threat (Lovibond, Mitchell, Minard, Brady, & Menzies, 2009). In other words, our model recasts “safety learning” and “fear extinction” as “repairing” inference. These constructs can thus be re-described as extending the counterfactual inference space to include hidden causes that are not threats (i.e., increased entropy).^5^

## Integration of free energy, molecular, neurodynamic, and connectivity models

Based on the account thus far, the dysfunction of PTSD can be associated with two distinct but intertwined phenomena. First, a PTSD-impaired individual may exhibit a higher prior bias for explaining sensory stimuli in terms of an existential threat, as compared to a healthy individual. In other words, in health, there may be occasions in which surprising stimuli do not necessarily indicate existential threat, and have alternative epistemic affordance (Figure 2, a_1_), whereas a PTSD response to the same surprising stimuli would amount to a threat identification and concomitant (in)action (Figure 2, b_1_).

Second, once a threat prior is operative, the scene resampling required for an assessment of the threat (Figure 2, c_3_) is compromised in PTSD (Figure 2, b_3_), such that any provisional threat identification is inevitably treated as confirmed and immutable (i.e., even in the absence of an actual threat, or if a momentary threat no longer persists). This amounts to saying that PTSD can be understood as a maladaptively prolonged threat-response state in the later absence of the original stressor that precipitated the condition. This offers an intuitive explanation for the combination of hyperarousal and hypocortisolaemia found in neuroendocrine studies of the hypothalamus-pituitary-adrenal (HPA) axis of PTSD patients (Sriram, Rodriguez-Fernandez, & Doyle, 2012; Yehuda, 2006).

The neurobiological substrate of strong top-down biases is usually associated with the encoding of precision in hierarchical (neuronal) message passing in the brain. This translates into setting the right postsynaptic gain—or sensitivity to ascending prediction errors—via mechanisms that generally involve neuromodulation (i.e., classical modulatory neurotransmitter systems or dynamical mechanisms such as synchronous gain). We can unpack the pathophysiology of this PTSD-compromised epistemic foraging in terms of aberrant neuromodulatory (precision) control of signals (e.g., prediction errors) that revise beliefs about states of the world, and the policies pursued. Stress drives Ca2+–cAMP signalling in the PFC, opening potassium (K+) ion channels (HCN, KCNQ), which leaves cells hyperpolarised, such that synaptic efficacy and network connectivity is weakened (Arnsten, 2009, 2015). This would preclude sensitive responses to ascending prediction errors, in that it would uncouple long-range signal propagation to higher cortical regions in exteroceptive hierarchies. Crucially, the latter are instrumental in stimulus comprehension, rather than mere detection (Khoshkhoo, Leonard, Mesgarani, & Chang, 2018).

Physiologically, setting the right sort of synaptic efficacy or postsynaptic gain can also be reflected in fast synchronised neuronal activity, implicating (e.g.) fast-spiking inhibitory interneurons (Bastos et al., 2012; Bosman et al., 2012; Chawla, Lumer, & Friston, 1999; Fries, 2005). In this setting, the healthy state is associated with gamma-frequency rhythms that both arise from, and facilitate, neuronal message passing and communication, while the absence of gamma-frequency rhythms is marked by sparser neuronal firing that results in lower information transfer across functionally defined neural regions (Fries, 2005). If, due to (e.g.) stress-induced cellular impairment, the brain cannot escape the (non-gamma) neuromodulator states that inhibit (re)sampling of the broader scene and the subsequent sensory sample disambiguation/integration processes, the remaining available perceptual behaviour (apart from complete shutdown or disorientation) would be computationally equivalent to underfitting—a functional decoupling of prior beliefs from adequately supportive sensory evidence about states of the world. In other words, if the neuronal circuitry has been “overloaded” by a prolonged threat response state, this state becomes a stronger attractor basin than that of the typical healthy state, resulting in PTSD. This is consistent with the observation that, when it would be adaptive to ascertain a subsequent threat-free scene and curtail the threat response state, in PTSD, the necessary neurochemical (glucocorticoid) “brake” to escape the HPA-related negative feedback loop is inhibited (Sherin & Nemeroff, 2011).

Further support for this overall picture is suggested by simulation results showing that stimulus-induced synchronous transients with strong propagation increasing mutual information across neuronal sub-populations is facilitated by fast synchronised activity (Chawla, Lumer, & Friston, 2000). Under the circumstances of a reduced number of neurons responding to restricted partial information from salient and background stimuli, there would be imprecise, impoverished sensory evidence, such that (Bayes) optimal responses would depend on high top-down priors, resulting in underfitting. We conjecture that, with this sort of pathophysiology, the top-down policy most readily available corresponds to the threat prior which, in this case, would be a strong association formed in the original circumstances that led to the PTSD (Tsoory et al., 2007). The threat prior would remain dominant, because exploratory sensory sampling and the evaluation of competing hypotheses would be physiologically impaired.

This threat association would accompany significantly amygdala-modulated behaviour, originally activated under the legitimate (pre-PTSD) existential threat condition (Tsoory et al., 2007), which appears to require only sparse information for activation (Figure 1c), in line with evolutionary considerations (Öhman, 2005). The amygdala-modulated subsystem activation pattern in turn inhibits the PFC-modulated subsystem that is active under safe conditions (Arnsten, 2009, 2015). On the above account, the latter (scene sampling) subsystem would still need to be intermittently activated under a threat condition, in order to ascertain that no threat persists by entertaining competing scene hypotheses (Figure 2, c_3_)—a process which would be impaired by PTSD (Figure 2, b_3_). Effective connectivity studies of PTSD patients suggest this aspect of impairment persists even in resting state, where otherwise regulatory connections are weakened (Nicholson et al., 2017).

In normal adaptation to danger, the amygdala is thought to play a role in associative learning and active hypothesis testing of ambiguous stimuli related to biologically relevant environmental events (Whalen, 1998). The response to danger can, however, become maladaptive such that it chronically impairs a number of regulatory processes (Rosen & Schulkin, 2004). It is this chronic impairment that gives rise to the range of distinctive PTSD maladaptations, in contrast to similarly underpinned healthy, adaptive responsiveness to stressors and traumatic events.

Consider findings by Öhman (2005), which demonstrated that low-threshold activation of the amygdala occurred during rapid fear-relevant stimulus presentation, but when

time was extended to allow conscious perception of the stimuli … there was strong bilateral amygdala activation to the actually feared stimuli (e.g., snakes), but no significant amygdala activation … in the fear-relevant but non-feared condition (e.g., spiders for a snake fearful participant).

We can relate this to healthy function and PTSD dysfunction, by noting that, to benefit from the longer time offered to healthy participants in the above study, the stimuli must be comprehended. This in turn depends upon long-range propagation to higher cortical areas (Khoshkhoo et al., 2018), for stimuli identified as non-threatening. Thus, if propagation is impaired by (e.g.) stress, there would be no ability for higher-order stimulus comprehension to ascertain that a perceived threat was misidentified, has subsided, or was neutralised. Instead, the runaway, self-affirming, top-down processes (i.e., policies) would continue to dominate sensory (exteroceptive) underfitting, by which sparse environmental samples underdetermine an incorrectly confirmed externally-originating threat. At the same time, (interoceptive) overfitting would treat all self-originating noise as evidence signalling an incorrectly justified feeling of endangerment.

This account is consistent with experiments showing that, even in the complete absence of a stimulus, the most frequently presented stimulus pattern across previous trials was (illusorily) perceived (Chalk et al., 2010). The latter implies that, once a strong prior is formed, it can contribute to high top-down bias with absent or weak sensory evidence (underfitting), as with a PTSD “flashback”, also referred to as an “intrusive” or “involuntary autobiographical” memory (Kvavilashvili, 2014), a quasi-hallucinatory state that differs from ordinary autobiographical recall (Brewin, 2015). Additional experimental evidence supports our model, in that PTSD flashbacks are associated with a decrement in visuospatial processing (Hellawell & Brewin, 2002), i.e., reduced exploration and integration in sensory sampling of the environment (epistemic foraging). Our integrative synthesis remains consistent with a wide range of clinical phenomena understood almost entirely independently of biology, from a systematic cognitive and behavioural perspective (Ehlers & Clark, 2000).

## Connections to related work

The present approach is continuous with the Bayesian computational neuropsychology of Parr, Rees, and Friston (2018), which considers inborn and lifespan neuronal loss, impairment, and paralysis arising in (e.g.) autism, neurodegenerative diseases, and physical brain damage. Here, we extend their account by the addition of experientially induced impairment (from trauma and stress) found in PTSD. Generally, on active inference accounts of psychopathology, sensory attenuation plays a significant role (Brown, Adams, Parees, Edwards, & Friston, 2013; Friston, 2017; Joyce, Averbeck, Frith, & Shergill, 2013; Oestreich et al., 2015; Parees et al., 2014; Shergill, Samson, Bays, Frith, & Wolpert, 2005). This is particularly relevant to our argument about PTSD, because the decoupling between sensory and higher-level neuronal activity, especially in the exteroceptive and interoceptive domains, can be described in terms of sensory attenuation—in other words, an attenuation of the influence that sensory information exerts over belief updating about policies. In predictive coding process theories, this decoupling would correspond to an attenuation of the precision afforded to ascending prediction errors.

Crucially, the PTSD story on offer in this paper is not simply a failure of sensory attenuation: on the current view, sensory attenuation in PTSD is mandated by selecting policies that involve action—namely, “fight”, “flight” or “freeze”. At a physiological level, it is as if one is confronted with a two-alternative forced choice between responding to and confirming a possible threat. The pathology here is not with sensory attenuation or neuromodulation *per se*, but rather, it rests on the inappropriate prior beliefs about policies that will minimise surprise in the future, based upon previous experience, where the default expectation is existential continuity. The final twist here is that once these policy responses to unconfirmed threats become established, they are self-maintaining. This is because they preclude alternative epistemic policies that would provide contradictory evidence, namely, to disconfirm the threat (cf. learned helplessness; Hammack, Cooper, & Lezak, 2012; Stephan et al., 2016).

### Trauma

A related account of stress, psychosis, and auditory hallucination is offered by Dodgson and Gordon (2009), who draw out cognitive and clinical connections to evolutionary threat detection function. As Wilkinson, Dodgson, and Meares (2017) point out, the former view is consistent with the Bayesian formalisms described by the predictive processing framework (Clark, 2016). In turn, further links between these accounts can be understood in terms of relationships between evolutionary biology, predictive coding, and active inference, as we have indicated above.

In their direct consideration of PTSD, Wilkinson et al. (2017) explore an established clinical typology of trauma. Type 1 trauma describes the experience of an acute, cataclysmic, life-threatening traumatic event (e.g., a terror attack), while Type 2 refers to the trauma of experiencing an extended period of threat (e.g., an abusive domestic relationship), both of which can lead to PTSD. They acknowledge that the type distinction can be blurred, and is not always able to be disentangled in clinical observation.

We map these types onto the present account, to suggest their distinctive underpinnings. Here, Type 1 trauma would be described as leading to a primed pathway for assigning a threat prior to a stimulus (Figure 2, b_1_). In the PTSD reaction, potentially relevant sensory evidence would be dampened, in favour of selecting a past-trauma associated model, driving an underfitting scenario with a functional decoupling from the PFC (Figure 1c). This would occur, for instance, when a bottle accidentally knocked off a shelf in a supermarket makes a loud breaking sound, which would lead to a (disproportionally frequent) immediate threat classification, in contrast to a healthy (balanced) probability of further scene sampling, to ascertain the possible misidentification of a threat. The dysfunctional response would in turn lead to the reflexive selection of a threat-responsive “fight or flight” policy.

Continuing with this account, Type 2 trauma would suggest the primed activation of a past-trauma associated model, such that it (disproportionately) frequently “wins” in model competition for stimulus-evoked scene recognition. Again, this stands in contrast to a healthy probability of further scene sampling, to ascertain misrecognition. In the PTSD reaction, if (for example) approaching footsteps are heard, these may be immediately classed as the approach of an abuser, evoking associations with the traumatic experience that has previously typically (serially) followed the sound of the abuser’s footsteps. In light of an approaching rather than currently present threat, instead of “fight or flight”, the approaching threat may plausibly lead to the selection of a “freeze” policy (mentioned above), a fearful, hypervigilant paralysis state, with simultaneous motor preparation and inhibition (Bracha et al., 2004; Roelofs, 2017).

Returning to our discussion of auditory masking, we can draw a direct correspondence between energetic masking and Type 1 trauma, and respectively, informational masking and Type 2 trauma. Essentially, events related to what are classed as Type 1 trauma appear to be commonly associated with high-energy auditory stimuli (e.g., explosions, gunfire, crashes, etc.), establishing an associative semantic and neuronally topographic link between such stimuli and an existential threat condition. Hence, a PTSD impairment would lead to model competition for maximising evidence for a threat condition, which would result in a “fight or flight” policy selection that redirects metabolic resources to the motor system (away from sensory exploration).

Given that Type 2 trauma is associated with an extended threat condition with a broader distribution of sensory stimulus intensities (e.g., living with an abuser), it is known that, for PTSD sufferers, even low-energy stimuli, such as quiet footsteps, can evoke episodes. In this case, model selection is biased toward a model that maximises evidence for an anticipated scenario in which the footstep sounds are regarded as precursors to an abuser’s infliction of acute trauma. This would allow even quiet footsteps to become “informational masks” that “capture attention” to the exclusion of other potentially salient information that indicates safe conditions (e.g., the sound of voices normally absent during abusive situations). Such model selection would also relate to the associative semantic and neuronally topographic links described above with respect to Type 1. In both cases, these links would relate to amygdala activation and PFC inhibition (Figure 1c).

### Context in context

Liberzon and Abelson (2016) offer a compelling synthesis and parsimonious generalisation and expansion of three predominant models of PTSD by appealing to a neurobiological account of contextual processing. On our view, this synthesis might be pushed further by situating neurobiological contextual processing within the broader scope of our explanatory stack. For instance, their description of a fundamental PTSD impairment in integrating “spatial properties of the environment … with non-spatial information (e.g., time, prior experience, and internal states) into a gestalt that becomes context” suggests a consilience in terms of the overall psychopathology (Liberzon & Abelson, 2016). Beyond the notion of a “context gestalt”, we propose that additional explanatory power is offered by understanding context in terms of the substrate-mapped formalism of a hierarchical Bayesian model architecture and attending neuronal process theories, performing what can be described as active inference and/or free energy minimisation (see e.g., Friston, 2008).

Similarly, we are in agreement that “dysfunction within … interconnected context processing circuitry—which involves hippocampus, prefrontal cortex, thalamus, and amygdala—may play a central role in the pathophysiology of PTSD” (Liberzon & Abelson, 2016). However, in place of a “just so” story about the neural circuitry involved in contextual awareness, additional explanatory power is offered by connecting neural structure and function to an ecologically situated, embodied and embedded cognitive architecture selected for by evolutionary pressures (Bracha et al., 2004; Clinchy et al., 2011; Dodgson & Gordon, 2009; LeDoux, 2012; Roelofs, 2017). Such pressures can be further contextualised in relation to fundamental energy-information trade-off requirements (Linson et al., 2018; Niven, 2016; Niven & Laughlin, 2008).

## Summary and conclusion

Based upon a conceptual analysis of the psychopathology and pathophysiology of PTSD, we have proposed an alternative perspective. We explored some implications of re-arranging the traditional or classical picture, in relation to top-down executive control, bottom-up sensory control, and attention, by recasting these in terms of continuous loops through an environmentally embedded, embodied neuronal architecture, and PTSD-related disruptions and alterations of these loops. Similarly, we presented an alternative account of neurocognitive reality testing, of generalisation of conditioned fear, fear extinction, and safety learning, as well as of informational and energetic auditory masking, and of contextual processing.

To illustrate multiscale and heterogeneous interactions, we formulated a multilevel explanatory stack. Our multilevel analysis can be summarised relatively straightforwardly, as follows: PTSD may induce prior beliefs that the appropriate policy—induced by sensory cues of uncertain origin—involves “fight or flight” or “freeze” responses, as opposed to exploratory active sensing (epistemic foraging). These maladaptive priors are particularly pernicious because they preclude the corrective steering function normally provided by sensorimotor interaction (“reality testing”), i.e., they inhibit the continuous testing of alternative hypotheses about states of the world that do not entail any existential threat. This leads to a biased (impoverished) sampling of the world and ultimately, a failure to revise the prior beliefs that underwrite pathological responses.

Physiologically, we consider this impaired belief updating to be a malignant form of sensory attenuation, namely, a diminishing of overt epistemic foraging in the exteroceptive domain, or a covert attenuation of the precision of exteroceptive signals. Both responses intrinsically conserve metabolic energy in environmental sensing, thereby making more available to policy-specific motor attention. Further metabolic reallocation arises from stopping short of sensory propagation to higher cortical areas, which may cease to be a readily available response due to neurochemical and sub-cellular imbalances that weaken effective connectivity.

In other words, in order to remain safe from existential threat, the PTSD response to surprising stimuli is all too frequently a physiological state change related to the colloquial expression “better safe than sorry”. In this state, when the question subsequently arises as to whether one can venture out to discover a possible “all clear”, any such venturing is thwarted by a secondary “better safe than sorry”, thereby remaining stuck in a self-maintaining threat preparedness state. This story appears to have predictive validity in relation to the known neurobiology, psychology, and behaviours associated with PTSD, and construct validity in relation to previous explanations for this, and related, disorders.

## Notes

1 This model can in turn be demonstrated using *in silico* simulations under the (Active Inference) Markov Decision Process scheme, which is the focus of a subsequent paper (Linson, Parr, & Friston, in preparation).

2 This stands in contrast to independent (i.e., non-interacting) “levels of explanation”, as in Marr-like decompositions, popular in cognitive science.

3 In brief, active inference can be regarded as a generalisation of e.g., reinforcement learning and optimal control theory, such that it replaces the optimisation of expected reward with the minimisation of expected surprise (where surprise can include negative rewards). Formally, active inference deals with the optimisations of (Bayesian) beliefs about hidden states and policies under uncertainty, enabling it to deal in a principled way with things like the exploration-exploitation dilemma. For example, it treats decision-making in terms of planning as inference. In this setting, free energy provides a bound upon surprise and is the negative of the evidence lower bound (ELBO) used in machine learning.

4 For an empirical account of these dynamics in terms of Global Neuronal Workspace Theory, see Vugt et al. (2018).

5 More broadly, the generative model entails counterfactual possibilities (of hidden causes) that can have different biases. When one possibility has a high bias, it can “win out” as the inferred hidden cause for a sense datum that, in reality, could have alternative hidden causes.
